# Identifying Some Risk Factors of Time to Relapses in Schizophrenic Patients using Bayesian Approach with Event-Dependent Frailty Model

**Published:** 2015-04

**Authors:** Maryam Rahmati, Mehdi Rahgozar, Farbod Fadaei, Enayatollah Bakhshi, Leila Cheraghi

**Affiliations:** 1 Department of Biostatistics, University of Social Welfare and Rehabilitation Sciences, Tehran, Iran; 2 Department of Psychiatry, University of Social Welfare and Rehabilitation Sciences, Tehran, Iran

**Keywords:** *Schizophrenia*, *Relapse*, *Recurrent Events*, *Event Dependence Frailty Model*, *Bayesian approach*

## Abstract

**Objectives:** Schizophrenia patients often experience relapses once and even more with no limit on number of relapses. The time among relapses are rarely considered in studies. The aim of this study was to identify some risk factors of time to elapses in schizophrenic patients with recurrent events model in survival analysis.

**Methods:** In a retrospective longitudinal study, the medical records of 159 schizophrenic patients who referred to Razi hospital in Tehran from 2003 to 2005 were conveniently sampled, investigated and followed until the end of 2009. The time to recurrent relapses were considered in weeks. The patients with at least one relapse in this duration were included in the study. Event-dependent frailty model, using Bayesian approach, was applied to fit the data and identify the risk factors of time to relapses.

**Results:** In this recurrent failure time model, the effects of age of onset (95% CI = (0.058, 0.086)), gender (95% CI = (0.146, 0.686)), marital status (95% CI = (0.475, 0.965)) and family history (95% CI = (0.115, 0.543)) were significant on the hazard time to relapses. According to the credible interval of frailty variance, elapsed time to relapse is dependent on patients’ characteristics (95% CI = (0.084, 0.369)). Subsequent relapses are likely to be influenced by the occurrence of the first relapse, too (95% CI = (2.504, 3.079)), with decreasing hazard of time to relapse.

**Conclusions:** Subsequent relapses are likely dependent on the first and previous relapses. Age of onset, gender, marital status and family history are important risk factors influencing hazard of time to relapses. More studies are required to clear out the effect of other covariates with this model.

Mental disorders have profound social, cultural and economic effects on communities worldwide (1). Burden of mental disorders are increasing markedly in developing countries. According to WHO reports, 12% of the global burden of disease is attributed to mental disorders, and it will be increased to 15% of disability-adjusted life years (DALYS) lost to illness by 2020 (2). There are a gamut range of patients of chronic mental disorder who could live in a normal situation, using maintenance medications (3). Schizophrenia is a mental illness that is among the world’s top ten causes of long-term disability. Delusions, hallucinations, bizarre behaviors, apathy and withdrawal and cognitive impairment are considered as symptoms of schizophrenia and result in problems in social and occupational functioning, and self-care. Lifetime prevalence of schizophrenia is about 1% regardless of nationality, culture and sex. 

The illness tends to develop between the ages of 16 and 30, and mostly persists in the course of patient’s lifetime. Although the causes of schizophrenia are unknown, evidences suggest that genetic factors, early environmental influences (e.g., obstetric complications), and social factors (e.g., poverty) play an important role (4).The incidence of schizophrenia indicates very important variation between sites. The median incidence of schizophrenia was 15.2/100,000 persons, and the central 80% of estimates varied over a fivefold range (7.7 – 43.0/100,000). The rate ratio of males to females was 1.4:1(5). There are considerable data of population-based studies on psychiatric morbidity in Iran, and many of past studies have been conducted in primary health care setting, or on specific population and disorders (6). One study in Iran has shown that the prevalence of schizophrenia is 0.25% and 0.13% and 0.18% for women and men, respectively (7). Age of onset and sex are documented to be fundamental to understanding schizophrenia (8). An earlier onset may be attributable to a genetic propensity to illness, as those with an earlier onset are more likely to have relatives with schizophrenia (9). Earlier onset is also associated with environmental risk factors, such as head injury before the age of 10 (10).Together, these genetic and environmental antecedents of onset may explain the clinical lure of age of onset (11). Evidence indicates that men have an earlier age of onset than women (4). Preventing relapse and thereby reducing the risk of unplanned acute readmissions is a very important goal in the treatment of patients with schizophrenia after discharge from hospital (12). Most schizophrenic patients have a chronic course with recurrent relapses characterized by exacerbation of psychosis and increase of re-hospitalization. An important feature of schizophrenia is the repetition of relapses over time (13). Analysis of time to relapse is important in the assessment of treatment outcome. Few studies have assessed time to relapse in patients with schizophrenia. However, most of them failed to account for correlations caused by recurrent events and event dependence which are key features of studies in schizophrenia. In this study, we present an event-dependent conditional frailty model using a Bayesian approach which separates and accounts for event dependence and heterogeneity in repeated relapses of schizophrenia. The aim was to identify some risk factors of time to relapses in schizophrenic patients with dependent recurrent events model in failure time data analysis using Bayesian approach.

## Material and Methods

*Participants and Procedure*

A retrospective longitudinal study was carried out on 162 conveniently sampled schizophrenic patients with at least one relapse, who were hospitalized at least once at Razi psychiatric hospital in Tehran between 2003 and 2005, and who were followed up until the end of 2009; data were collected from their medical records. Three patients who had missing values on all covariates in their records were excluded from the total sample. For the rest of the patients, the onset of schizophrenia was considered as the time of their follow up, and they were followed in terms of their relapses. Relapse was defined as observing schizophrenia symptoms in the first re-hospitalization after discharge and following re-hospitalizations. We assessed time to relapses and the number of weeks patients remained to be re-hospitalized. Some patients right censored at their last time period to re-hospitalize. 

*Statistical Analysis*

 Recurrent time data are commonly encountered in longitudinal studies when failure events can occur repeatedly over time for each study subject (14). Time to relapses in schizophrenic patients can be studied with suitable failure time models. A common feature for these times is the correlations that occur naturally in a certain sequence over time (15). There are two sources of correlation in such recurrent events like relapses. The first is event dependence in which the second and subsequent relapses are likely to be influenced by the occurrence of the first and previous relapses. The second source is heterogeneity and occurs when some subjects are more prone to experience recurrent relapses than others (16). Ignoring these sources of correlations will result in underestimating standard errors of estimates for effects of covariates (17). We considered these two sources of correlations as relapse dependence and modeled as frailty effect. Methods such as Cox proportional hazard for failure time data could not be used here because they do not take into account such correlation structures in the data (18).We applied Weibull Conditional hazard frailty model which separates and accounts for event dependence and heterogeneity in recurrent events data like relapses. To infer about the hazard function and model parameters of relapses times, we considered relapse or re-hospitalization events and elapsed time after each discharge to readmission as a gap time. The period of treatment (hospitalization) was excluded since when patients were admitted, they were treated and cared during the period of hospitalization. Since the next recurrence risk of relapse for the patients may reduce, these patients may be at risk of having a relapse again upon hospital discharge. For each participant, the first gap time is defined as the time elapsed between onset and the first hospitalization; the second gap time is the elapsed time from the first release till the second hospitalization; the third gap time is the elapsed time from the second release till the third hospitalization and so on. The Bayesian approach was used to estimate the parameters of the model. The advantage of this model is that it gives not only point estimates for unknown parameters by incorporating prior information in a natural way but also for entire posterior distributions for model parameters, making inference easier and more precise than classic statistical method. It also enables us to make precise inferences for any sample size without resorting to asymptotic assumptions (16,18). Available data fields included age at onset, gender, marital status, mode of onset, history of head injury and family history of schizophrenia. Win BUGS software was used to analyze data.

## Results

The participants for the studied sample were 134 (84.28%) males and 25 (15.72%) females. The mean age of the patients at the onset of the disease was 21.52 ± 6.84 years (range 10-43). The relative frequency of married patients was 19.59% (31), and 85 (53.46%) patients experienced sudden onset of disease while 76 (47.8% ) patients had a history of head injury. Only 10 (6.29%) patients had schizophrenic patients in their family (grades one to three kinship). In common survival models, positive signs of regression coefficients increase the risk of event occurrence. 

**Table 1:** Frequency of Relapses in Schizophrenic Patients due to their Gender and Family History

		** Number of Relapses**			
Family History	Gender	2	3	4	5 and more
Yes	Male Female	0 0	3 (50.0%) 1 (33.3%)	2 (33.3%) 2 (66.7%)	1 (16.7%) 0
No	Male Female	4 (3.1%) 0	58 (45.3%) 14 (63.6%)	30 (23.4%) 6 (27.3%)	36 (28.1%) 2 (9.0%)

**Table 2:** Posterior Summaries for the Parameters of Weibull Frailty Model

**Variable**		**Reference** **category**	**Mean**	**Standard deviation**		**95% Credible** **Interval for HR**
Age of onset		-	0.072		0.007	(0.058 , 0.086)
Gender	Male	Female	0.418		0.137	(0.146 , 0.686)
Marital status	Single	Married	0.710		0.125	(0.475 , 0.965)
Mode of onset	Gradual	Sudden	0.199		1.111	(-0.019 , 0.412)
History of head injury	Yes	No	0.285		0.234	(-0.165 , 0.757)
Family history	Yes	No	0.327		0.109	(0.115 , 0.543)
Ѕ*			2.785		0.147	(2.504 , 3.079)
ϒ+			0.860		0.0334	(0.796 , 0.926)
Frailty variance			0.206		0.0721	(0.084 , 0.369)

* The effect of event dependence

+ Shape parameter of Weibull distribution

Distribution of the number of relapses in schizophrenic patients shows that 45.3% of males who did not have a family history of schizophrenia had experienced 3 relapses during the study period (Table 1). With respect to their credible intervals, age of onset (95% CI = (0.058 , 0.086)), gender (95% CI = (0.146 , 0.686)), marital status (95% CI = (0.475 , 0.965)) and family history (95% CI = (0.115 , 0.543)) had significant effects on the conditional hazard function and increased the risk of relapses considering their positive coefficients. Using the posterior means, the estimate of event dependence parameter ( ) was 2.785 with (95% CI (2.504, 3.079)) and of the shape parameter ( ) of Weibull distribution was 0.860 with (95% CI = (0.796, 0.926)) which were significant according to their credible intervals (Table 2). We can conclude that second and subsequent relapses are likely to be influenced by the occurrence of the first and previous relapse. This implies a decrease in hazard. The posterior frailty variance, which measures the degree of heterogeneity among subjects and the correlation among relapses times was estimated to be 0.206 with (95% CI = (0.084, 0.369)) (Table 2). Since the lower bound of the interval was not zero, there was heterogeneity among the participants.

## Discussion

Chronic diseases such as schizophrenia are, roughly speaking, lifelong transitions between the states of relapse and recovery. The long-term pattern of recurrent times-to-relapse can be investigated with routine register data on hospital admissions. The relapses indicate the time for readmissions to hospital and the time spent at the hospital are considered gaps between subsequent times-at-risk (19). In this study, Bayesian analysis of Weibull conditional hazard frailty model with event-dependent effect was developed to estimate the parameters. In the model, the effects of age of onset, gender, marital status and family history were significant. Wang and Chang used a nonparametric estimation for the marginal survival function of the gap time and demonstrated that the onset age of schizophrenia plays an important role in determining the length of time between hospitalizations (20). Chang used a regression analysis for the gap time and considered both discharged and readmitted events in schizophrenia and showed that age of onset has a significant effect on the average duration of remission but no effect on length of hospital stay (14). A testing procedure was proposed and applied to schizophrenia data set by Chen and Wang to detect whether or not there was a progressive trend in the gap times of hospitalization. They found deterioration patterns of the disease among the patients (21). In contrast to their findings, Chen et al. showed that both male and female patients share similar deterioration patterns, but it was not significant in male patients. For married patients, the deterioration pattern was significant while it was not as significant among the unmarried (22). Mortensen, et al., using data from the civil registration system in Denmark, showed that although a history of schizophrenia in a parent or sibling is associated with the highest relative risk of having the disease, the place and season of birth account for many more cases on a population basis (23). Nielsen, et al. examined the rates of head injury among 8288 persons in the 15 years up to their first admission with schizophrenia and compared them with 82880 age and gender-matched controls, using hospitalization for concussion or severe head injury. In their research, no difference were found in severe head injury (24). Xiang, et al. investigated the relapse rate and its socio-demographic and clinical correlates and predictors in Chinese schizophrenic patients following the treatment of the acute phase of the illness. Their study confirmed the importance of maintenance medication in preventing relapse in Chinese schizophrenic patients. They also showed that socio-demographic characteristics were not associated with relapse (25). Using recurrent events model, Rahgozar, et al. illustrated that history of head injury and gender were significant on the hazard rate of relapses; they considered only the first three relapses in their study (26). In this study, the result revealed that the variance of frailty was significant. Olesen and Mortensen analyzed the schizophrenia data in a shared frailty model adjusting for calendar year, age of onset, duration of latest hospital admission as confounders and estimated a frailty variance of 0.448, which was highly significant, suggesting that considerable heterogeneity was present (27).We concluded that the shape parameter of Weibull distribution was less than 1 which resulted in decreasing hazard. Robinson, et al. concluded that there was a high rate of relapse within 5 years of recovery from a first episode of schizophrenia. This risk is diminished by maintenance antipsychotic drug treatment (28).

## Conclusion

Subsequent relapses are likely dependent on the first and previous relapses. Age of onset, gender, marital status and family history are important risk factors influencing hazard of time to relapses. More studies are required to clear out the effect of other covariates such as place of residence (urban vs. rural), education level, economic status and season of birth of patients on time to relapses with this model.
